# A radio-pathological fusion model for predicting PD-L1 expression and immunotherapy response in non-small cell lung cancer

**DOI:** 10.1186/s13244-026-02322-4

**Published:** 2026-06-15

**Authors:** Dingpin Huang, Fangyi Xu, Yi Gan, Liya Ding, Kaihua Lou, Yongcen Li, Dong Xie, Haiping Zhang, Lei Shi, Rui Xu, Hongjie Hu

**Affiliations:** 1 Department of Radiology, Zhejiang Cancer Hospital, Hangzhou Institute of Medicine (HIM), Chinese Academy of Sciences, Hangzhou, Zhejiang China; 2 Department of Radiology, Sir Run Run Shaw Hospital, Zhejiang University School of Medicine, Hangzhou, Zhejiang China; 3 Medical Imaging International Scientific and Technological Cooperation Base of Zhejiang Province, Sir Run Run Shaw Hospital, Zhejiang University School of Medicine, Hangzhou, Zhejiang China; 4 Department of Pathology, Sir Run Run Shaw Hospital, Zhejiang University School of Medicine, Hangzhou, Zhejiang China; 5 DUT-RU International School of Information Science and Engineering, Dalian University of Technology, Dalian, Liaoning China; 6 DUT-RU Co-Research Center of Advanced ICT for Active Life, Dalian University of Technology, Dalian, Liaoning China; 7 Department of Radiology, Shaoxing Second Hospital, Shaoxing, China

**Keywords:** Non-small cell lung cancer, Immunotherapy, PD-L1, Radiomics, Pathology

## Abstract

**Objective:**

This study aims to construct a multimodal fusion model (FM) based on CT and hematoxylin and eosin (H&E) stained slices to predict the PD-L1 expression in non-small cell lung cancer (NSCLC) and to explore its additional value in predicting the prognosis of immunotherapy.

**Materials and methods:**

A retrospective analysis was conducted of 328 NSCLC patients with available PD-L1 immunohistochemical results. They were randomly divided into a training set, a validation set, and a test set in a 4:1:1 ratio. Radiomics and pathological models were constructed based on CT images and H&E slides, respectively, to predict PD-L1 expression, and then a radio-pathological FM was established. Then, the radio-pathological FM was used to generate predictive scores for an independent NSCLC immunotherapy survival validation cohort.

**Results:**

A total of 55.5% (182/328) of patients were PD-L1 positive and included in the PD-L1 prediction cohort. Compared to the single-modality model, the radio-pathological FM achieved the highest predictive performance, with AUCs of 0.90, 0.80, and 0.73 across the three subsets, respectively. In the survival validation cohort, patients in the high-score group had significantly better progression-free survival (PFS) and overall survival than those in the low-score group. Furthermore, the FM score was an independent predictor of PFS. When combined with clinical factors, its *C*-index for predicting PFS was 0.74 (95% CI: 0.665–0.809).

**Conclusion:**

For the first time, a radio-pathological FM was constructed to predict PD-L1 expression in NSCLC. The study also demonstrated the model’s potential for predicting patient prognosis under immunotherapy.

**Critical relevance statement:**

This first fusion model combining CT radiomics and hematoxylin and eosin (H&E) deep learning non-invasively predicts programmed death-ligand 1 (PD-L1) and immunotherapy response in non-small cell lung cancer (NSCLC).

**Key Points:**

- The fusion model can accurately predict programmed death-ligand 1 (PD-L1) and immunotherapy outcomes in non-small cell lung cancer (NSCLC).
- The fusion model outperformed either single-modality model in distinguishing PD-L1-positive.
- Potential to reduce PD-L1 immunohistochemical testing and support precision immunotherapy decisions.

**Graphical Abstract:**

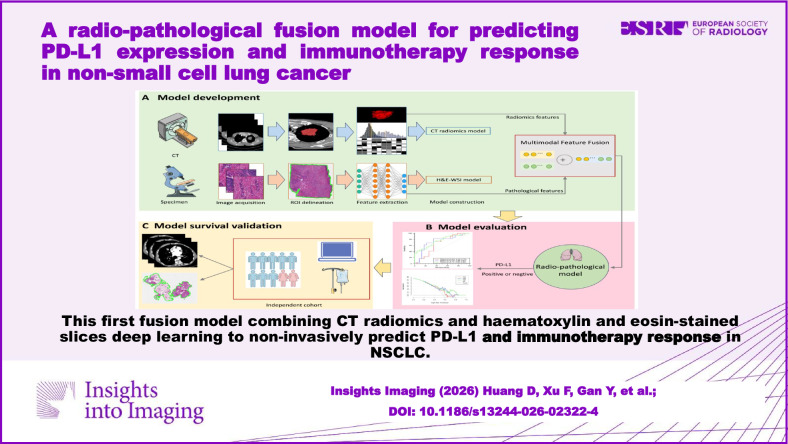

## Introduction

Lung cancer has become the leading cause of cancer-related deaths worldwide [1]. Immune checkpoint inhibitors (ICIs) have shown significant survival benefits in patients with advanced or metastatic non-small cell lung cancer (NSCLC) [2–4]. However, immunotherapy is not suitable for all lung cancer patients. Sometimes, tumors do not respond to immunotherapy [5], and immune-related adverse events are also common [6]. Therefore, identifying which NSCLC patients will benefit from immunotherapy is crucial. Several large multicenter clinical studies have shown that NSCLC patients with Programmed Death-Ligand 1 (PD-L1) positive expression are more likely to benefit from ICIs than those with negative expression [7, 8]. However, PD-L1 detection is usually based on immunohistochemical (IHC) staining of specimens, and many shortcomings remain to be addressed in clinical practice [9]. Firstly, there are multiple staining platforms for PD-L1 detection, resulting in highly variable evaluation criteria [10]. Secondly, PD-L1 expression levels are subject to subjective pathologist evaluation, which is more likely to introduce bias [11].

Radiomics contains both the features of the entire tumor, such as first-order, morphological, and texture features, and the spatial features of the tumor, which intuitively reflect the information of the surrounding structures [12]. However, this reflection is indirect, and a significant gap persists between radiomic features and the tumor tissue's actual histological characteristics. The hematoxylin and eosin (H&E) stained slides of tumors contain a large amount of microscopic information, including tumor cell characteristics, density, tumor microenvironment, and other information related to tumor invasiveness [13, 14]. Furthermore, whole-slide images (WSI) contain a large amount of hidden information and provide a reliable basis for quantitative analysis of histopathological slides [15]. The features combined from CT and H&E-stained slides are a multi-scale fusion of macroscopic and microscopic information, which can more comprehensively reflect tumor information and accurately evaluate tumor heterogeneity and prognosis [16–18].

Thus, we hypothesize that a fusion model (FM) based on CT and H&E-stained slides has complementary information, which is beneficial for more accurate PD-L1 expression evaluation. To the best of our knowledge, no such attempt has been made thus far. Therefore, in this study, we constructed a radiomics model (RM) based on CT images and a pathological deep learning model based on H&E-WSI, respectively, to predict PD-L1 expression (positive or negative) in NSCLC. Subsequently, we fused the features of the two to establish a radio-pathological FM and validated it in an independent NSCLC cohort receiving immunotherapy to explore its additional value in predicting immunotherapy outcomes.

## Methods

### Study population

**Fig. 1:**
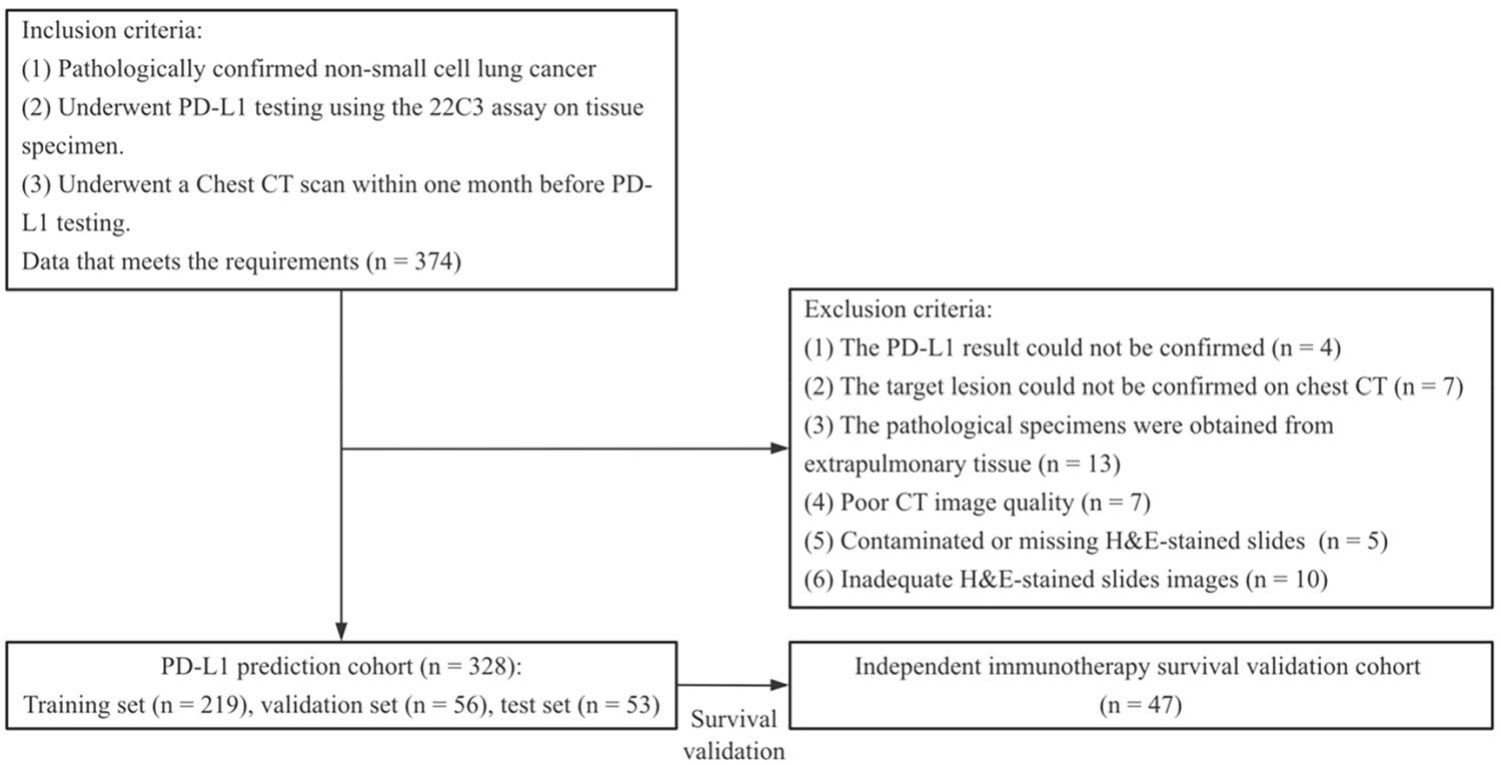
Flowchart of patient inclusion and exclusion in this study. PD-L1, programmed death-ligand 1

This study was approved by the ethics committee of Sir Run Run Shaw Hospital (No. 2022-0432). Informed consent was waived because the study was retrospective. The PD-L1 expression prediction model was developed using data from patients diagnosed with NSCLC in the hospital from January 2020 to July 2022, with PD-L1 results. The inclusion criteria were as follows: (1) pathologically confirmed NSCLC; (2) underwent PD-L1 testing using the 22C3 assay on tissue specimens; (3) underwent a chest CT scan within one month before PD-L1 testing. Patients were excluded if any of the following conditions were met: (1) the PD-L1 result could not be confirmed; (2) the target lesion could not be confirmed on chest CT; (3) the pathological specimens were obtained from extrapulmonary tissue; (4) poor CT image quality (e.g., motion artifacts); (5) contaminated or missing H&E-stained slides; (6) inadequate H&E-stained slides images, due to issues such as tissue folding, poor staining, or insufficient tumor content. The detailed process of patient inclusion and exclusion is shown in Fig. 1.

To verify the additional value of the radio-pathological FM in predicting immunotherapy prognosis, we retrospectively enrolled NSCLC patients who underwent immunotherapy. Inclusion criteria for this cohort were as follows: (1) pathologically confirmed NSCLC from June 2017 to October 2019; (2) received monotherapy with ICIs or combination immunochemotherapy for more than 2 cycles; (3) availability of histological specimens and chest CT images obtained within one month before treatment; (4) H&E-WSI and CT images met the requirements of experimental analysis; (5) availability of clinical and follow-up survival data for analysis.

According to RECIST 1.1 criteria [19], progression-free survival (PFS) is defined as the time from the initiation of immunotherapy to the first documented disease progression or death from any cause. Disease progression is defined as a ≥20% increase in the diameter of the target lesion, with an absolute increase of ≥5 mm. Overall survival (OS) is defined as the time from the initiation of immunotherapy to death from any cause. The final follow-up date was August 10, 2024. A total of 47 patients were included in the immunotherapy survival validation cohort.

### Pathological evaluation

The PD-L1 IHC testing in this study was performed using the Dako 22C3 antibody on the Dako Autostainer Link 48 platform. All slides were formalin-fixed paraffin-embedded (FFPE). Two senior pathologists (Dr. Y. Gan and Dr. L.Y. Ding, each with more than 10 years of experience) independently evaluated PD-L1 expression in tumor cells. The tumor proportion score (TPS) was used to represent the PD-L1 expression of the tumor [10, 19]. PD-L1 positive was defined as TPS ≥ 1%, while PD-L1 negativity was defined as TPS < 1%. In cases of disagreement between the two pathologists regarding PD-L1 expression levels, consensus was reached through discussion. Corresponding H&E-stained slides were scanned at 40x magnification using a digital pathology scanner (KF-PRO-400, KFBIO) for data analysis.

### Construction of a radiomics model

All patients underwent chest CT examination prior to PD-L1 testing. Detailed CT scan and reconstruction parameters are provided in Part I and Part II of the Supplementary Material. All CT images were imported into ITK-SNAP software (Version 3.8.0). An experienced radiologist (Dr. D.P. Huang, with over 10 years of experience) manually delineated the tumor ROI slice by slice, blinded to the pathological results. To assess segmentation consistency, 30 cases were randomly selected and independently re-segmented 1 month later by Dr. D.P. Huang and Dr. K.H. Lou (a radiologist with over 2 years of experience). Inter- and intra-class correlation coefficients (ICCs) were calculated to assess the reproducibility of radiomics features within and between observers. An ICC ≥ 0.85 was considered indicative of good reproducibility [20].

Radiomics features—including shape, first-order, and texture features—were extracted using the PyRadiomics package. For feature selection, t-tests and least absolute shrinkage and selection operator (LASSO) regression were used to identify the most discriminative radiomics features. Using the final set of stable features, models were built using five classifiers: decision tree, Gaussian process, support vector machine, logistic regression, and random forest. The classifier with the best performance on the test set was selected for final prediction. After evaluation using ICCs, t-tests, and LASSO regression, 19 stable features were selected for model training (Supplementary Table S2). A logistic regression classifier was used to build the final model.

### Construction of a pathological deep learning model

First, H&E image data were preprocessed. For each WSI, foreground and background areas were segmented using Otsu’s method. Subsequently, the WSI was divided into patches of size 1792 × 1792 pixels using a sliding window approach. Second, a pre-trained ResNet-50 model was used to extract features from the image patches within each WSI. Given that deep layers of neural networks capture high-level semantic information related to the dataset, whereas shallow layers capture low-level feature representations of the input data, feature extraction was performed using the third block of the ResNet model. The output of the third block was saved as the extracted features for subsequent model training.

In this study, the pathology stream was implemented using a dual-stream multiple instance learning (DSMIL) framework [21], which operates at the WSI level and does not require patch-level annotations. Each WSI was divided into multiple non-overlapping patches, and the model was trained using only the slide-level PD-L1 TPS label, defined as positive or negative. Within the DSMIL architecture, an attention mechanism automatically assigned a weight to each patch based on its relevance to the WSI-level prediction. These learned attention scores reflect the model’s internal estimate of how likely each patch is to contribute to a positive classification. A weighted sum of all patch features, guided by these attention scores, was then used to form a unified WSI-level representation, which was passed to a final fully connected classifier. Importantly, no patch-level labels were generated, inferred, or used during training; all supervision came exclusively from the WSI-level ground truth.

### Construction of radio-pathological fusion model

**Fig. 2:**
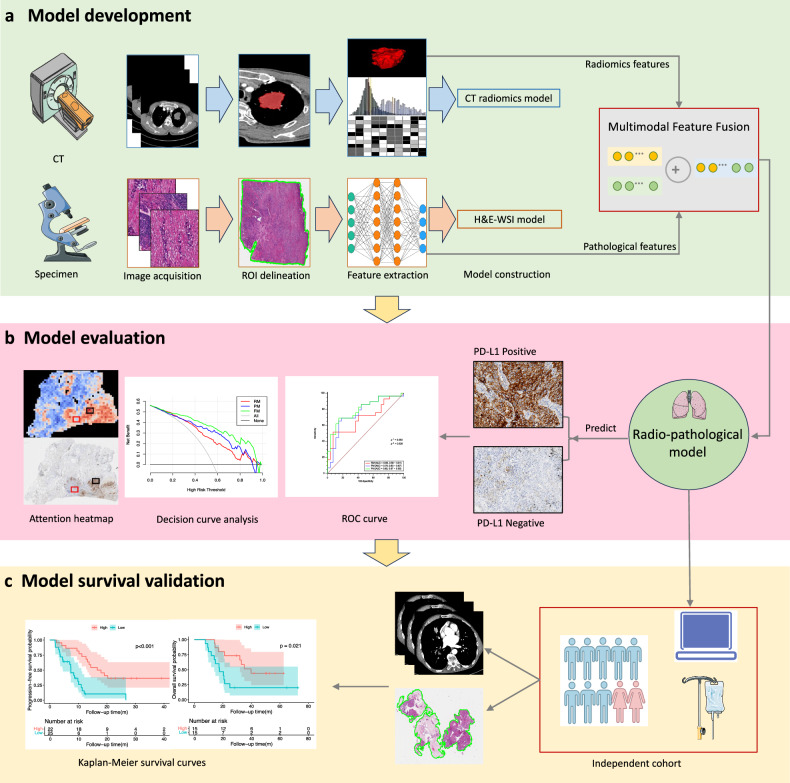
Study workflow. **a** We used pre-treatment CT and pathological specimens for feature delineation and extraction to construct a CT radiomics model, a pathological deep learning model, and a radio-pathological fusion model for predicting PD-L1 expression. **b** Model performance was evaluated, and the fusion mechanism was visualized using attention maps. **c** The fusion model was validated in an independent immunotherapy survival validation cohort to assess its ability to predict survival outcomes. H&E, hematoxylin and eosin; PD-L1, Programmed Death-Ligand 1; ROC, receiver operating characteristic; WSI, whole-slide images

In this study, we integrated radiomics features from CT images and deep-learning pathological features from H&E-stained slices of the same patients. The workflow of the study is illustrated in Fig. 2.

Radiomics features were represented by a 20-dimensional vector, and pathological features by a 768-dimensional vector. Prior to fusion, both feature sets were independently normalized using min-max scaling to align their value ranges between 0 and 1. No dimensionality reduction was applied to either modality. The normalized radiomics and pathological features were then directly concatenated to form a single 788-dimensional multimodal feature vector for each patient. This fused vector was fed into a linear classification layer, with model training supervised by cross-entropy loss.

### Statistical analysis

Statistical analyses were conducted using SPSS (Version 25.0), MedCalc (Version 20.1), and RStudio (Version 2023.12.1). Continuous variables were assessed for inter-group differences using independent-samples *t*-tests or the Mann–Whitney *U-*test. Categorical variables were assessed for inter-group differences using chi-square tests or Fisher’s exact tests. The performance of the predictive models was evaluated using receiver operating characteristic (ROC) curves, with the area under the curve (AUC) calculated and compared using the DeLong method. The clinical utility of the predictive models was assessed using decision curve analysis (DCA).

The cutoff values for the radio-pathological FM scores associated with survival outcomes were calculated using X-tile software (Version 3.6.1, Yale University School of Medicine). Kaplan-Meier survival curves and the log-rank test were used to compare PFS and OS in the immunotherapy validation cohort. Univariate and multivariate Cox regression analyses were applied to identify factors associated with survival, and the C-index was used to evaluate the predictive ability of these factors for survival outcomes. A *p*-value < 0.05 was considered statistically significant.

## Results

### PD-L1 prediction cohort

A total of 328 patients were enrolled and randomly divided into a training set (*n* = 219), a validation set (*n* = 56), and a test set (*n* = 53) in a nearly 4:1:1 ratio. The final cohort included 182 PD-L1 (+) and 146 PD-L1 (−) patients. Baseline characteristics of the PD-L1 prediction cohort are presented in Tables 1 and 2.

**Table 1:** Clinical characteristics of the PD-L1 prediction cohort

Characteristics	Total (%) (*n* = 328)	PD-L1 (+) (*n* = 182)	PD-L1 (-) (*n* = 146)	*p*-value
Sex				0.048*
Male	261 (79.6)	152 (83.5)	109 (74.7)	
Female	67 (20.4)	30 (16.5)	37 (25.3)	
Age (years)	67 (59,73)	68 (61,73)	66 (57,73)	0.184
Smoking history				0.535
Current or before	141 (43.0)	81 (44.5)	60 (41.1)	
Never	187 (57.0)	101 (55.5)	86 (58.9)	
CEA (ng/mL)	4.43 (2.26–9.61)	4.66 (2.37–9.94)	3.83 (2.16–8.72)	0.519
Histology				0.203
Adenocarcinoma	142 (43.3)	71 (39.0)	71 (48.6)	
Squamous	159 (48.5)	94 (51.6)	65 (44.5)	
Others	27 (8.2)	17 (9.3)	10 (6.8)	
Tumor stage				0.036*
I	48 (14.6)	19 (10.4)	29 (19.9)	
II	23 (7.0)	10 (5.5)	13 (8.9)	
III	121 (36.9)	69 (37.9)	52 (35.6)	
IV	136 (41.5)	84 (46.2)	52 (35.6)	

Data are expressed as median (range) or number (percentage). *p < 0.05

*CEA* carcinoembryonic antigen, *PD-L1* Programmed Death-Ligand 1

**Table 2:** Clinical characteristics of the training set, validation set, and test set

Characteristics	Training set (*n* = 219)			Validation set (*n* = 56)			Test set (*n* = 53)			
	PD-L1(+) (*n* = 123)	PD-L1(−) (*n* = 96)	*p-*value	PD-L1(+) (*n* = 29)	PD-L1(−) (*n *= 27)	*p-*value	PD-L1(+) (*n* = 30)	PD-L1(−) (*n* = 23)	*p-*value	*p-*value^#^
Sex			0.034*			0.227			0.679	0.548
Male	104 (84.6)	70 (72.9)		26 (89.7)	21 (77.8)		22 (73.3)	18 (78.3)		
Female	19 (15.4)	26 (27.1)		3 (10.3)	6 (22.2)		8 (26.7)	5 (21.7)		
Age (years)	68 (62,74)	67 (57,73)	0.113	67 (59,72)	65 (58,71)	0.687	66 (62,70)	68 (59,74)	0.660	0.505
Smoking history			0.702			0.945			0.528	0.503
Current or before	57 (46.3)	42 (43.8)		11 (37.9)	10 (37.0)		13 (43.3)	8 (34.8)		
Never	66 (53.7)	54 (56.2)		18 (62.1)	17 (63.0)		17 (56.7)	15 (65.2)		
CEA (ng/mL)	4.44 (2.41,9.94)	3.73 (2.17, 7.81)	0.293	5.12 (2.59,15.36)	3.42 (1.67,8.72)	0.342	4.52 (1.94,7.11)	5.37 (3.41,40.25)	0.132	0.814
Histology			0.215			0.865			0.946	0.422
Adenocarcinoma	45 (36.6)	46 (48.0)		13 (44.8)	14 (51.9)		13 (43.3)	11 (47.8)		
Squamous	68 (55.3)	45 (46.9)		12 (41.4)	10 (37.0)		14 (46.7)	10 (43.5)		
Others	10 (8.1)	5 (5.2)		4 (13.8)	3 (11.1)		3 (10.0)	2 (8.7)		
Tumor stage			0.050			0.065			0.929	0.965
I	14 (11.4)	18 (18.8)		1 (3.4)	7 (25.9)		4 (13.3)	4 (17.4)		
II	6 (4.9)	11 (11.5)		3 (10.3)	1 (3.7)		1 (3.3)	1 (4.3)		
III	44 (35.8)	35 (36.5)		13 (44.8)	7 (25.9)		12 (40.0)	10 (43.5)		
IV	59 (48.0)	32 (33.3)		12 (41.4)	12 (44.4)		13 (43.3)	8 (34.8)		

Data are expressed as median (range) or number (percentage). *p < 0.05

*p*-value^#^: Comparison of characteristics among the three sets

*CEA* carcinoembryonic antigen, *PD-L1* programmed death-ligand 1

The majority of patients were male (79.6%), with a median age of 67 years, and 43% had a history of smoking. Statistically significant differences were observed between the PD-L1 (+) and PD-L1 (−) groups for sex and tumor stage, whereas no significant differences were observed for age, smoking history, carcinoembryonic antigen (CEA), or pathological type. No significant differences in clinical characteristics were observed among the three subsets: the training, validation, and test sets.

### Performance of different models

**Fig. 3:**
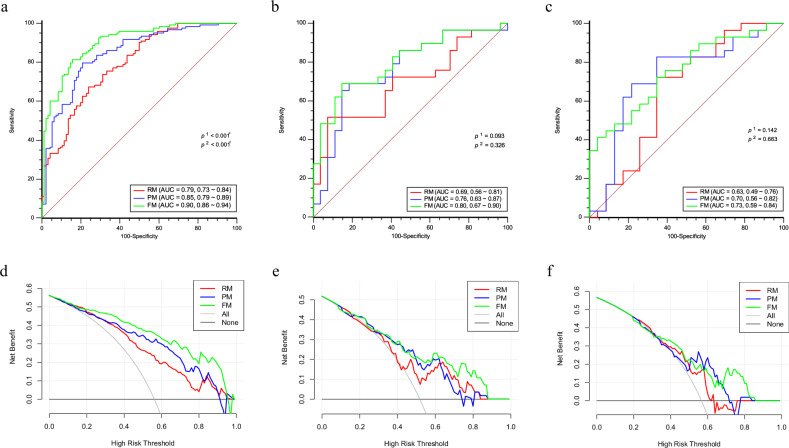
The predictive performance of different models. The AUCs of different models in **a** training, **b** validation, and **c** test sets. The decision curve analysis for the different models in **d** training, **e** validation, and **f** test sets. AUC, area under the curve; RM, radiomics model; PM, pathological model; FM, fusion model

**Fig. 4:**
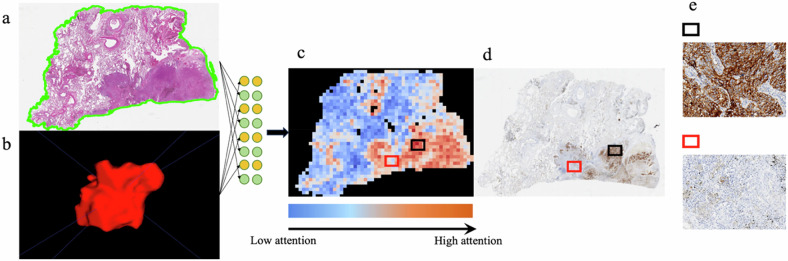
The attention visualization heatmap of the fusion model. A 65-year-old male was diagnosed with lung adenocarcinoma and was positive for PD-L1 (TPS = 80%). By integrating the **a** H&E features and **b** CT features, **c** an attention heatmap of the fusion model was obtained. It can be observed that the fusion model focused its attention on most of the stained tumor cells (the red area indicates that the model has more attention, whereas the blue area indicates that the model pays less attention), similar to the **d** IHC image. **e** The black box indicates that most tumor cells were stained by the 22C3 antibody, while the red box indicates that most tumor cells were not stained. H&E, hematoxylin and eosin; IHC, immunohistochemical; PD-L1, Programmed Death-Ligand 1; TPS, tumor proportion score

The RM achieved AUCs of 0.79, 0.69, and 0.63 in the training, validation, and test sets, respectively. The pathological model (PM) yielded AUCs of 0.85, 0.76, and 0.70 across the three sets. The FM demonstrated the highest predictive performance, with AUCs of 0.90, 0.80, and 0.73 in the respective subsets. ROC curves and associated metrics are presented in Fig. 3a–c and Table 3. DeLong’s test revealed that, in the training set, the FM significantly outperformed both the radiomics and PMs in distinguishing PD-L1 positive from negative expression (*p* < 0.001). However, in the validation and test sets, although the AUC of the FM was numerically higher, the differences were not statistically significant (*p* > 0.05) (Table 3). Decision curve analysis demonstrated that, across all three sets, the FM provided greater net benefit across a wide range of threshold probabilities compared to the other two models (Fig. 3d–f). To visualize the decision-making mechanism of the FM, an attention heatmap was generated. It shows that the model correctly attended to most PD-L1-stained tumor cells, consistent with high PD-L1 expression (Fig. 4).

**Table 3:** The predictive performance and parameters of different models

		Radiomics	Pathomics	Fusion model	*p*-value^a^	*p*-value^b^
Training set	AUC [95%CI]	0.79 [0.73, 0.84]	0.85 [0.79, 0.89]	0.90 [0.86, 0.94]	< 0.001*	< 0.001*
	Specificity	0.76	0.72	0.66		
	Sensitivity	0.68	0.72	0.92		
	Accuracy	0.71	0.74	0.80		
	Precision	0.74	0.76	0.77		
	F1 score	0.74	0.74	0.84		
Validation set	AUC [95%CI]	0.69 [0.56, 0.81]	0.76 [0.63, 0.87]	0.80 [0.67, 0.90]	0.093	0.326
	Specificity	0.70	0.69	0.56		
	Sensitivity	0.57	0.69	0.83		
	Accuracy	0.64	0.70	0.70		
	Precision	0.69	0.71	0.67		
	F1 score	0.68	0.70	0.74		
Test set	AUC [95%CI]	0.63 [0.49, 0.76]	0.70 [0.56, 0.82]	0.73 [0.59, 0.84]	0.142	0.663
	Specificity	0.63	0.71	0.44		
	Sensitivity	0.53	0.71	0.83		
	Accuracy	0.58	0.72	0.66		
	Precision	0.57	0.71	0.66		
	F1 score	0.62	0.71	0.74		

*p*-value: The AUCs of different models were compared using DeLong’s test

^a^ *p* value^1^: Radiomics vs. Fusion model

^b^ *p* value^2^: Pathomics vs. Fusion model

* *p* < 0.05

*AUC* area under the curve, *CI* confidence interval

In addition to the primary analysis based on PD-L1 TPS ≥ 1%, we evaluated the model’s ability to identify patients with high PD-L1 expression (TPS ≥ 50%). On the test set, the FM achieved an AUC of 0.69 (95% CI: 0.55–0.81), with a sensitivity of 75.0% and specificity of 59.5% for detecting TPS ≥ 50% (Supplementary Fig. S1). While performance was lower than that observed with the ≥ 1% threshold, the relatively high sensitivity suggests potential utility for ruling out low PD-L1 expressors or for prioritizing cases for confirmatory IHC testing.

### Validation of prognostic prediction of the radio-pathological fusion model

**Fig. 5:**
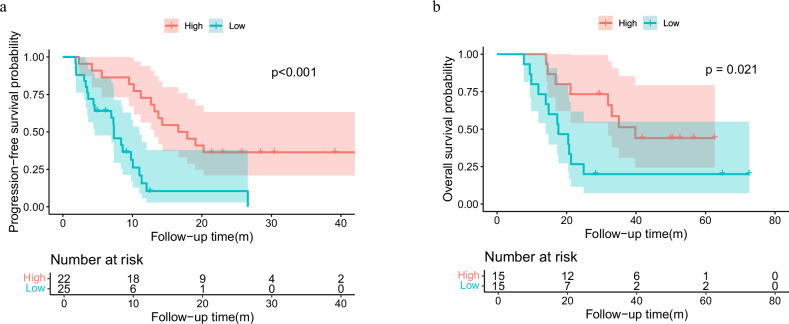
Kaplan–Meier survival curves of the immunotherapy survival validation cohort. It indicated both **a** PFS and **b** OS were significantly better in the high-score group compared to the low-score group. OS, overall survival; PFS, progression-free survival

A total of 47 patients were included in this cohort after applying the inclusion and exclusion criteria (Table 4). The majority were male (93.6%), with a median age of 67 years, and squamous cell carcinoma was the predominant histological type (59.6%). Among them, 21 (44.7%) received monotherapy with ICIs, and 26 (55.3%) received combination immunochemotherapy. The median PFS for this cohort was 8.34 months. For OS, 17 patients were lost to follow-up, and the median OS among the remaining 30 patients was 29.47 months. The radio-pathological FM was used to generate a score for each patient. Using X-tile software, the optimal cutoff value for the FM score in predicting survival prognosis was determined to be 0.46. Based on this cutoff, patients were stratified into a high-score group (≥ 0.46) and a low-score group (< 0.46), and differences in clinical characteristics between the two groups were assessed (Table 4). Univariate analysis revealed no significant differences between the groups for most clinical features. However, the median PFS was significantly longer in the high-score group compared to the low-score group (16.6 vs. 7.33 months, *p* < 0.001), and median OS was also significantly longer (39.77 vs. 17.63 months, *p* = 0.021). Kaplan–Meier survival curves confirmed significant differences in both PFS and OS between the two groups (Fig. 5), with superior outcomes in the high-score group, suggesting that the FM score has significant prognostic value in immunotherapy. Further stratified survival analyses by treatment regimen, histological subtype, and clinical stage consistently demonstrated that the fusion model score was predictive of PFS across subgroups, whereas its association with OS remained limited—likely due to incomplete follow-up and small sample sizes in subgroup comparisons (Supplementary Fig. S2). Univariate Cox regression analysis (Table 5) identified smoking history (*p* = 0.045, HR = 2.00), combination immunochemotherapy (*p* = 0.015, HR = 0.42), and FM score (*p* < 0.001, HR = 0.0006) as factors associated with PFS. Multivariate Cox regression analysis (Table 5) confirmed that both combination immunochemotherapy and FM score were independent predictors of PFS (*p* < 0.05). The *C*-index for the clinical model incorporating smoking history and treatment regimen was 0.63. When the FM score was added, the predictive accuracy improved (*C*-index: 0.74, 95% CI: 0.67–0.81).

**Table 4:** Clinical characteristics of the independent immunotherapy survival validation cohort

Characteristics	Total (*n* = 47)	Fusion model score		
		High-score group (*n* = 22)	Low-score group (*n* = 25)	*p*-value
Sex				0.909
Male	44 (93.6)	20 (90.9)	24 (96.0)	
Female	3 (6.4)	2 (9.1)	1 (4.0)	
Age (years)	67 (60–72)	67 (62–72)	66 (60–73)	0.701
Smoking history				0.681
Current or before	22 (46.8)	11 (50.0)	11 (44.0)	
Never	25 (53.2)	11 (50.0)	14 (56.0)	
CEA (ng/mL)	4.50 (2.80–9.60)	4.07 (2.51–6.71)	4.77 (2.92–12.82)	0.247
Histology				0.390
Adenocarcinoma	13 (27.7)	4 (18.2)	9 (36.0)	
Squamous	28 (59.6)	15 (68.2)	13 (52.0)	
Others	6 (12.8)	3 (13.6)	3 (12.0)	
Tumor stage				0.949
III	19 (40.4)	9 (40.9)	10 (40.0)	
IV	28 (59.6)	13 (59.1)	15 (60.0)	
Treatment regimen				0.626
Mono-I	21 (44.7)	9 (40.9)	12 (48.0)	
Comb-IC	26 (55.3)	13 (59.1)	13 (52.0)	
Median PFS	11.00	16.60	7.33	< 0.001*
(95% CI)	(7.33, 13.73)	(11.20, 20.23)	(4.40, 10.07)	
Median OS	21.27	39.77	17.63	0.021*
(95% CI)	(17.30, 39.77)	(16.83,39.77)	(9.83, 21.27)	
NA	17 (36.2)	7 (31.8)	10 (40.0)	

Data are expressed as median (range) or number (percentage). *p < 0.05

*CEA* carcinoembryonic antigen, *CI* confidence interval, *Comb-IC* combination immunochemotherapy, *Mono-I* monotherapy with ICIs, *NA* not available*, OS* overall survival, *PFS* progression-free survival

**Table 5:** Univariate and multivariate Cox regression analyses and *C*-index of the independent immunotherapy survival validation cohort

Characteristics	Univariate			Multivariate			
	HR (95%CI)	*p*-value	*C*-index (95% CI)	HR (95%CI)	*p*-value	*C*-index (95%CI)^a^	*C*-index (95%CI)^b^
Sex (male)	0.87 (0.27,2.88)	0.825	0.51 (0.49, 0.53)				
Age	1.03 (0.99,1.07)	0.193	0.59 (0.49, 0.69)				
Smoking history (current or before)	2.00 (1.02,3.93)	0.045*	0.59 (0.51, 0.67)	1.75 (0.87, 3.51)	0.115		
CEA	1.00 (0.99,1.00)	0.336	0.58 (0.34, 0.50)				
Histology (adenocarcinoma)			0.55 (0.46, 0.64)				
Squamous	0.82 (0.37, 1.80)	0.618					
Others	1.07 (0.36,3.21)	0.905					
Tumor stage (IV)	1.12 (0.57,2.21)	0.737	0.51 (0.43, 0.59)				
Treatment regimen (Comb-IC)	0.42 (0.21,0.84)	0.015*	0.59 (0.52, 0.67)	0.35 (0.17, 0.73)	0.005*	0.63 (0.54, 0.73)	
Fusion model score	0.0006 (0.00, 0.05)	< 0.001*	0.70 (0.64,0.76)	0.0002 (0.00, 0.02)	< 0.001*		0.74 (0.67, 0.81)

* *p* < 0.05

*CEA* carcinoembryonic antigen, *CI* confidence interval, *Comb-IC* combination immunochemotherapy, *HR* hazard ratio

^a^ The *C*-index for the clinical predictive factors integrating smoking history and treatment regimen

^b^ The *C*-index for combining smoking history, treatment regimen, and the fusion model score

## Discussion

Currently, several biomarkers have been established as predictors of immunotherapy response, including PD-L1, tumor mutation burden (TMB) [22], and circulating tumor DNA (ctDNA) [23]. However, the detection methods for these biomarkers differ, and their predictive performance varies significantly [24, 25]. Among them, PD-L1 remains the first and most widely used biomarker in clinical practice, despite its suboptimal predictive accuracy. Nevertheless, PD-L1 assessment via IHC testing has several limitations—such as spatial heterogeneity, inter-observer variability, and sampling bias—that may compromise the reliability of the results. In this study, we pioneered the integration of radiomics with deep learning analysis of histopathological images to construct a radio-pathological FM for predicting PD-L1 expression in NSCLC. Importantly, our findings further demonstrate that this multimodal model holds promise for predicting survival outcomes following immunotherapy.

Our results showed that the RM achieved AUCs of 0.79, 0.69, and 0.63 in the training, validation, and test sets, respectively. Radiomics, by enabling non-invasive extraction of macroscopic spatial and textural features from medical images, has demonstrated substantial clinical value in disease diagnosis, treatment guidance, and prognosis prediction [12]. In contrast, histopathological images provide detailed information on tumor cellular morphology and the tumor microenvironment, offering direct insights into tumor invasiveness and heterogeneity [26]. Numerous studies have confirmed that deep learning-based analysis of pathology images can effectively predict gene mutations and treatment responses [14, 27, 28]. Consistently, our pathological deep learning model achieved AUCs of 0.85, 0.76, and 0.70 across the three datasets. Notably, the PM outperformed the RM across all subsets, likely because histopathological data more directly capture cellular and microenvironmental features relevant to PD-L1 expression and immunotherapy response. Therefore, integrating radiomic and pathological information may enhance the dimensionality and biological relevance of predictive models, enabling more accurate assessment of tumor biomarkers and therapeutic outcomes.

By combining CT-derived radiomics features and H&E-based pathological features, the radio-pathological FM effectively distinguished PD-L1-positive from PD-L1-negative NSCLC patients. The model achieved AUCs of 0.90, 0.80, and 0.73 in the three subsets, demonstrating superior performance compared to either single-modality model. Notably, our cohort for model development included 328 patients—smaller than those in other published radiomics studies [29–31]. Despite this relatively limited sample size, our model achieved competitive predictive accuracy. This may be attributed to the complementary nature of multimodal data: the integration of macroscopic imaging with microscopic pathological details provides rich, multi-scale biological information that compensates for data scarcity. These findings underscore the potential of multimodal fusion to enhance model performance even with smaller datasets, highlighting the scientific rigor and innovation of our approach.

Furthermore, the FM demonstrated prognostic value in predicting outcomes for NSCLC patients receiving immunotherapy. Patients with high FM scores exhibited significantly better PFS and OS than those with low scores, suggesting its clinical utility in guiding treatment decisions for advanced NSCLC. Subgroup analyses revealed that the model score had consistent predictive value for PFS across different clinical and pathological subgroups, whereas its ability to predict OS was less robust. This discrepancy may be explained by the more objective nature of PFS assessment, while OS is influenced by multiple factors—including subsequent therapies, drug resistance, comorbidities, and socioeconomic status [32]. Additionally, 17 patients (approximately 36.2%) were lost to follow-up, resulting in incomplete OS data and potentially attenuating the model’s predictive power for OS. Univariate and multivariate Cox regression analyses confirmed that both combination immunochemotherapy and the FM score were independent predictors of PFS, further supporting its incremental prognostic value.

A major limitation of artificial intelligence in medicine is the “black box” nature of many models, which limits transparency and clinical trust [33]. To improve interpretability, we generated attention heatmaps to visualize the regions the FM focused on during prediction. These heatmaps revealed that the model predominantly attended to tumor cells with membranous PD-L1 staining, while non-stained tumor cells and non-tumor stromal cells received less attention. This visual evidence supports the biological plausibility and specificity of the model’s predictions, reducing concerns about arbitrary or spurious associations.

Another key innovation of our study lies in its clinical feasibility. Although PD-L1 IHC remains the clinical gold standard, it has well-recognized limitations, including tissue consumption, inter-observer variability, and occasional equivocal results, particularly near clinically relevant thresholds such as 1%. Our FM addresses these challenges by using data that are routinely available before treatment: CT scans and H&E-stained WSI. Importantly, H&E slides are generated as part of the standard pathology workflow in all patients, so the model provides PD-L1-related insights without requiring additional tissue or a dedicated IHC test. Therefore, the model is not intended to replace IHC, but to serve as a complementary tool in specific clinical situations. For example, it may be helpful when tissue is insufficient for comprehensive biomarker testing, when IHC results are borderline or delayed, or when early risk stratification is needed to guide treatment discussions. By leveraging existing diagnostic materials, this approach has the potential to streamline decision-making, reduce costs, and lessen patient burden.

Several limitations should be acknowledged. First, this is a retrospective study, which inherently carries a risk of selection bias. Second, this study has a relatively small sample size and a single-center design, which may affect the generalizability of the model. The performance drop from training to the test set suggests some degree of overfitting, which may be attributed to the high dimensionality of pathological features and limited sample size. Notably, the immunotherapy validation cohort is small, and OS data were incomplete due to loss to follow-up, limiting the robustness of survival analyses. Accordingly, our primary clinical claims are based on PFS, for which data are more complete, while conclusions regarding OS are presented as exploratory. And the lack of external validation further limits generalizability. Future studies with larger, multi-center cohorts are needed to validate and refine the model. Third, there is notable heterogeneity in the data. CT scans were acquired using different scanners and protocols, while pathological specimens included both biopsy and surgical samples. In addition, response assessment was based on RECIST 1.1 rather than iRECIST due to the limited availability of confirmatory scans in routine practice. While this may underestimate true immunotherapy-related progression patterns, it reflects real-world constraints and is consistent with many retrospective biomarker studies. Finally, some patients were lost to follow-up in the survival cohort, which may have introduced bias in OS estimation and limited the model’s ability to predict long-term outcomes.

In summary, this study presents the first radio-pathological FM that integrates CT imaging and H&E pathology to predict PD-L1 expression in NSCLC. We further demonstrate its potential value in predicting immunotherapy prognosis. These findings support the feasibility and promise of multimodal AI models in precision oncology.

## ELECTRONIC SUPPLEMENTARY MATERIAL

Supplementary Material

## Data Availability

The datasets used and/or analyzed during the current study are available from the corresponding author upon reasonable request.

## Abbreviations

AUC: Area under the curve
CEA: Carcinoembryonic antigen
CI: Confidence interval
Comb-IC: Combination of immunochemotherapy
FM: Fusion model
H&E: Hematoxylin and eosin
HR: Hazard ratio
IHC: Immunohistochemical
Mono-I: Monoimmunotherapy
NA: Not available
NSCLC: Non-small cell lung cancer
OS: Overall survival
PD-L1: Programmed death-ligand 1
PFS: Progression-free survival
PM: Pathological model
RM: Radiomics model
TPS: Tumor proportion score
WSI: Whole-slide images
