# Supplementary material for: A radio-pathological fusion model for predicting PD-L1 expression and immunotherapy response in non-small cell lung cancer
Source: Insights Imaging. 2026 Jun 15;17:161. doi: 10.1186/s13244-026-02322-4 (PMC13269606; doi:10.1186/s13244-026-02322-4)
Supplement: Supplementary file 1 — Supplementary Material [file 13244_2026_2322_MOESM1_ESM.pdf]

A radio-pathological fusion model for predicting PD-L1 expression and immunotherapy response in non-small cell lung cancer

ELECTRONIC SUPPLEMENTARY MATERIAL

I. CT image acquisition

II. Table S1 Scanning parameters and CT specifications

III. Table S2 The selected radiomics features

IV. Figure S1 Performance of Fusion Model for predicting PD-L1 TPS ≥ 50% on the test set

V. Figure S2 Stratified analysis for high-score and low-score groups in different subgroups

I. CT image acquisition

All patients underwent chest CT examination, and the CT equipment and scanning parameters are shown in Supplementary Table S1. For contrast-enhanced scans, non-ionic contrast agents (Ultravist 300 or Ultravist 370, Bayer; or Ioversol 320, Hengrui) were administered intravenously at a dose of 1.2 mL/kg body weight and an injection rate of 2.2 to 3 mL/s. Bolus tracking technology was utilized, initiating the arterial phase scan 8 seconds after the CT value in the descending aorta reached 100 HU. All CT scans were reconstructed using standard reconstruction kernels at the mediastinal window settings with a thickness of ≤ 2mm. CT data were retrieved from the picture archiving and communication system (PACS) for further feature extraction.

II. **Table S1** Scanning parameters and CT specifications

Brand	Siemens	Siemens	Siemens	Siemens	GE	GE
Machine type	SOMATOM Definition Flash	SOMATOM Force	SOMATOM Definition AS	SOMATOM go. Top	Lightspeed VCT	Optima CT620
Tube voltage (KV)	120	100/120	120	120	120	120
Tube current (mAs)	smart	smart	smart	smart	smart	smart
Rotation time (s)	0.5	0.5	0.5	0.5	0.4、0.5	0.5
Image matrix (mm)	512 × 512	512 × 512	512 × 512	512 × 512	512 × 512	512 × 512
Field of view (mm)	350	350	350	350	350	350
Reconstruction slice thickness and spacing	2 mm/2 mm	2 mm/2 mm	1 mm/1 mm 2 mm/2 mm	2 mm/2 mm	1.25 mm/ 1.25 mm	2 mm/2 mm
Reconstruction algorithm	B41f	B41f	B41f	B41f	Standard resolution	Standard resolution

III. Table S2 The selected radiomics features

Feature types	No.	Feature name
Original texture features	1	original_GLCM_Autocorrelation
	2	original_GLCM_ClusterShade
	3	original_GLCM_InverseVariance
High-order features of Log transformation	4	log-sigma-1-0-mm-3D_GLCM_ClusterShade
	5	log-sigma-1-0-mm-3D_GLSZM_GrayLevelNonUniformityNormalized
	6	log-sigma-3-0-mm-3D_GLCM_ClusterShade
	7	log-sigma-3-0-mm-3D_GLCM_Imc2
	8	log-sigma-3-0-mm-3D_GLSZM_GrayLevelVariance
	9	log-sigma-3-0-mm-3D_GLSZM_SmallAreaLowGrayLevelEmphasis
High-order features of Wavelet transformation	10	wavelet-LLH_GLSZM_LargeAreaLowGrayLevelEmphasis
	11	wavelet-LLH_GLDM_LargeDependenceHighGrayLevelEmphasis
	12	wavelet-LHH_GLSZM_LargeAreaLowGrayLevelEmphasis
	13	wavelet-HLH_firstorder_Mean
	14	wavelet-HLH_firstorder_Skewness
	15	wavelet-HLH_GLCM_Imc1

	16	wavelet-HLH_GLDM_LargeDependenceHighGrayLevelEmphasis
	17	wavelet-HHH_GLCM_ClusterShade
	18	wavelet-HHH_GLCM_Imc2
	19	wavelet-LLL_firstorder_Kurtosis

IV. Figure S1 Performance of Fusion Model for predicting PD-L1 TPS ≥ 50% on the test set

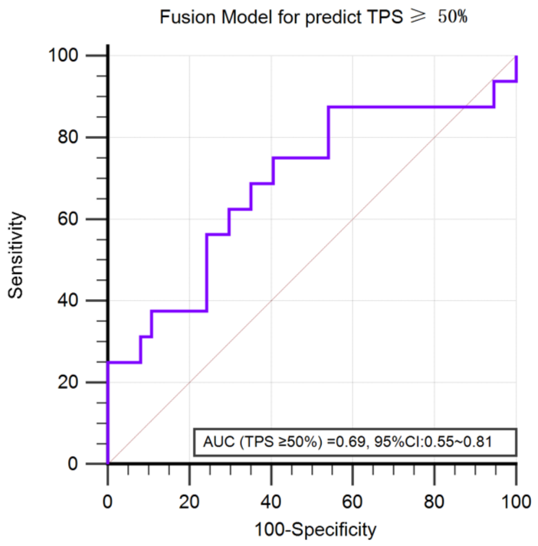

Figure S1

Performance of Fusion Model for predicting PD-L1 TPS ≥ 50% on the test set (number of TPS ≥ 50% cases: n = 13). Achieving an AUC of 0.69 (95% CI: 0.55-0.81), with a sensitivity of 75.0% and specificity of 59.5%.

IV. Figure S2 Stratified analysis for high-score and low-score groups in different subgroups

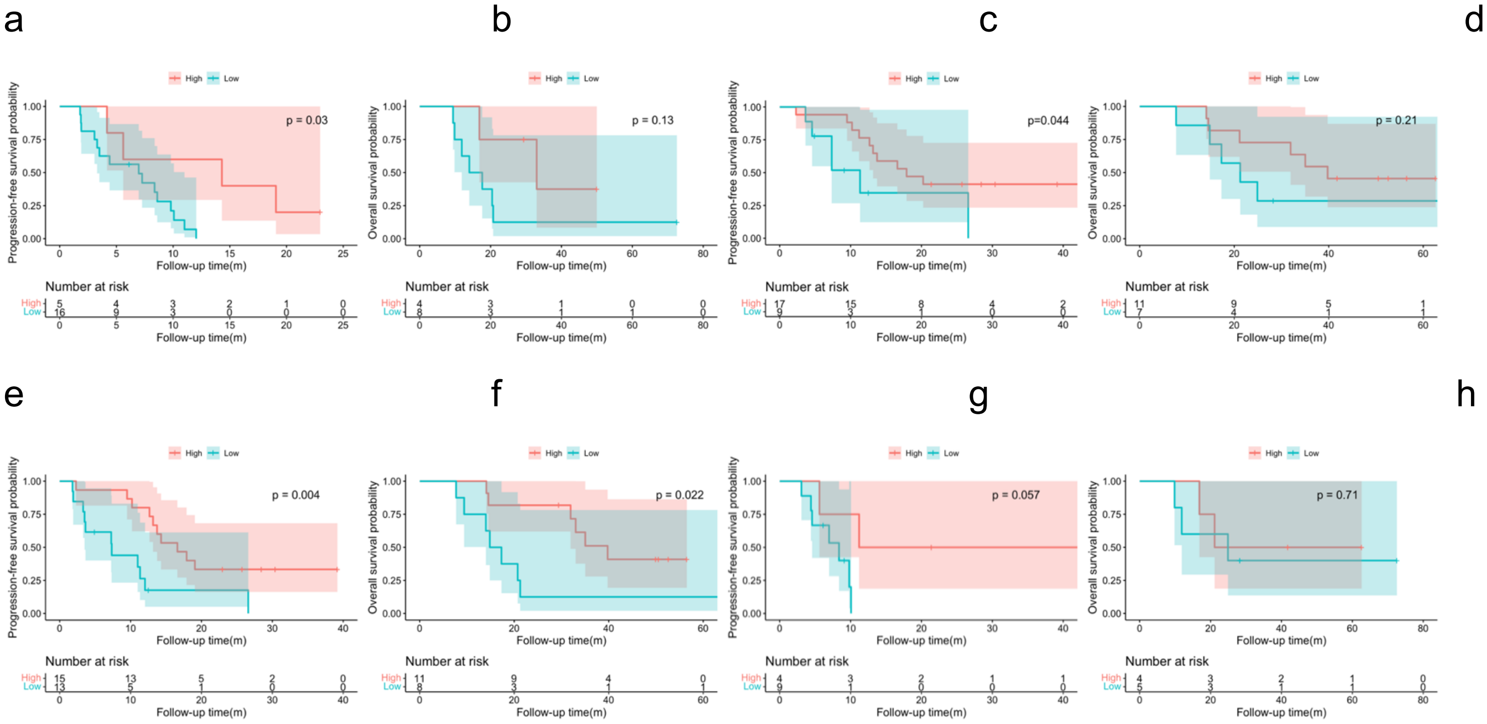

i j k l

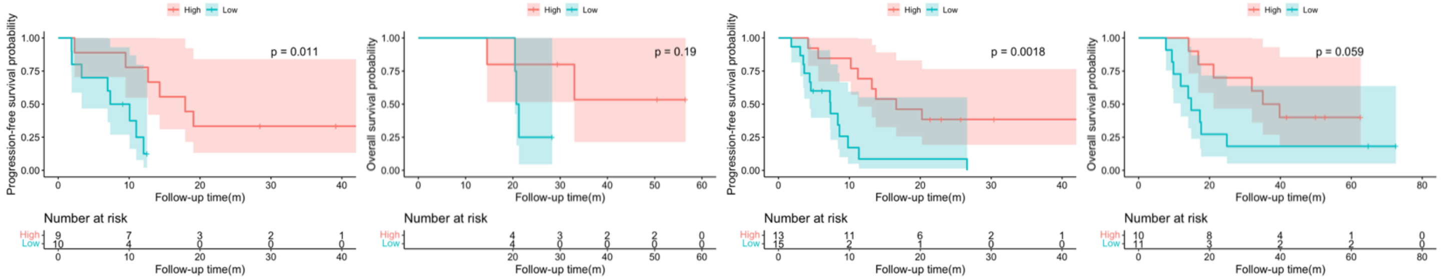
High
Low
p = 0.011
Progression-free survival probability
Follow-up time(m)
Number at risk
p = 0.19
Overall survival probability
p = 0.0018
p = 0.059

Figure S2

(a~d) Subgroup analysis for different treatment regimens. (a, c) PFS of the high-score group was significantly higher than that of the low-score group whether patients received monotherapy with ICIs or combination immunochemotherapy. (b, d) For OS, there was no significant difference between the two groups.

(e~h) Subgroup analysis for different histologies. (e~f) PFS and OS of the high-score group in squamous carcinoma patients were significantly better than that of the low-score group. (g~h) For adenocarcinoma patients, there were no significant difference in PFS and OS between the high-score group and low-score group.

(i~l) Subgroup analysis for different tumor stages. (i, k) PFS of the high-score group was significantly higher than that of the low-score group for both stage III and stage IV NSCLC patients. (j, l) For OS, there was no significant difference between the two groups.
